# The diagnostic value of white blood cell, neutrophil, neutrophil-to-lymphocyte ratio, and lymphocyte-to-monocyte ratio in patients with primary angle closure glaucoma

**DOI:** 10.18632/oncotarget.16571

**Published:** 2017-03-25

**Authors:** Shengjie Li, Wenjun Cao, Jianping Han, Binghua Tang, Xinghuai Sun

**Affiliations:** ^1^ Department of Clinical Laboratory, Eye & ENT Hospital, Shanghai Medical College, Fudan University, Shanghai, China; ^2^ Department of Ophthalmology & Visual Science, Eye & ENT Hospital, Shanghai Medical College, Fudan University, Shanghai, China; ^3^ State Key Laboratory of Medical Neurobiology, Institutes of Brain Science and Collaborative Innovation Center for Brain Science, Fudan University, Shanghai, China; ^4^ Key Laboratory of Myopia, Ministry of Health, Fudan University, Shanghai, China; ^5^ Shanghai Key Laboratory of Visual Impairment and Restoration, Fudan University, Shanghai, China

**Keywords:** primary angle closure glaucoma, white blood cell, neutrophil, neutrophil-to-lymphocyte ratio, lymphocyte-to-monocyte ratio

## Abstract

**Objective:**

Inflammatory mechanisms may have a role in the pathogenesis of primary angle closure glaucoma (PACG). The objective of this study was to investigate the diagnostic value of white blood cell (WBC), neutrophil, neutrophil to lymphocyte ratio (NLR), and lymphocyte to monocyte ratio (LMR) in patients with PACG and its association with glaucoma severity.

**Method:**

The study was retrospectively assessed in 771 consecutive PACG patients and 770 control subjects, laboratory parameters and clinical parameters were obtained from a medical data platform. Patients were divided into three groups with different severity based on perimetry, i.e. mild (mean deviation (MD) ≤6.00 dB), moderate (12 dB≥ MD>6 dB) and severe (MD>12 dB). We developed a nomogram to specifically identify individual patient’s risk.

**Results:**

The mean levels of neutrophil, NLR and WBC was higher in PACG than control group, and lowest in the mild PACG group, followed by moderate PACG and severe PACG (*p*<0.05). The AUROC value of NLR and LMR was found to be 0.719, 0.699, respectively. Multiple linear regressions showed that there was a significant correlation between WBC and MD (B=0.151, *p*<0.001), neutrophil and MD (B=0.143, *p*=0.003), NLR and MD (B=0.144, *p*=0.001), LMR and MD (B=-0.100, *p*=0.034). Logistic regression analyses revealed that WBC (OR=1.208, 95%CI=1.179-1.238), neutrophil (OR=1.598, 95%CI=1.541-1.656), NLR (OR=2.313, 95%CI=2.200-2.431), and LMR (OR=0.682, 95%CI=0.666-0.699) were associated with PACG.

**Conclusion:**

Our study suggested that WBC, neutrophil, NLR, and LMR was related with PACG, and NLR and LMR may be useful as biomarkers.

## INTRODUCTION

Primary glaucoma represents a major public health burden with unknown cause characterized by the degeneration of retinal ganglion cells and their axons associated with progressively irreversible blindness. This is especially so with a rapidly aging global society and the relatively high prevalence of glaucoma being observed among the elderly [1, 2]. Primary angle closure glaucoma (PACG) is a major subgroup of glaucoma in China. There is increasing evidence that autoimmune, active infection and inflammatory mechanisms may have a role in the pathogenesis of glaucoma [3–8], but the details of this relationship are yet to be established precisely. Zeng et al. [7] reported that infection of H. pylori have a statistically significant association with glaucoma. In addition, Gramlich et al. [5] also found that retinal IgG autoantibody were at least twice as high as in healthy subjects and CD27(+) cells and CD27(+)/IgG(+) plasma cells were observed in all glaucomatous subjects. Moreover, our previous studies found that the level of complement C3 was decreased in patients with PACG [9]. These studies provide critical information that there is a potential role for inflammation as an initiating or exacerbating factor in some patients with PACG, and this should be further assessed.

A systemic inflammatory response in PACG will increase the circulating counts of white blood cell (WBC), neutrophil, monocyte, and platelet. The neutrophil-to-lymphocyte ratio (NLR), platelet-to-lymphocyte ratio (PLR) and lymphocyte-to-monocyte ratio (LMR), are simple and inexpensive methods for assessing inflammation, which have been investigated as predictors of several cancers [10, 11], cardiovascular [12] and inflammatory diseases [13]. However, to the best of our knowledge, there are only two papers which reported that NLR and PLR may be useful as biomarkers in patients with primary open-angle glaucoma [14] and pseudoexfoliation glaucoma [15]. Studies on the association between WBC, neutrophil, monocyte, platelet, NLR, PLR, and LMR with PACG are limited.

The purpose of this study was to analyze the value of the counts of peripheral WBC, neutrophil, monocyte, lymphocyte, NLR, PLR, and LMR in a large series of patients with PACG and investigate the value of NLR, PLR, and LMR levels in predicting the diagnosis of patients with PACG as simple and easily accessible indicators of inflammation.

## RESULTS

### Characteristics of the study population

A total of 771 subjects with PACG (females = 498, males = 273) and 770 normal controls (females = 513, males = 257) were enrolled in this study. Only one eye was selected randomly if both eyes suffered from PACG. A total of 771 eyes from the PACG group were randomized. The demographic and laboratory parameters between PACG and control group are summarized in Table 1. There was no statistical difference in the mean age and gender between PACG and control subjects (*p* > 0.05). A significant difference was found in terms of WBC, PLT, neutrophil, lymphocyte, monocyte, NLR, PLR and LMR levels between PACG and control group, with *p*-values of < 0.001.

**Table 1:** Comparison of demographic and laboratory parameters between PACG and control groups

Factors	PACG group (*N*=771)	Control group (*N*=770)	*P* value
Age (years)	63.43±10.68	63.35±10.55	0.875
Gender(Male/Female)	273/498	257/513	0.401
WBC (10^9^/L)	6.19±1.90	5.87±1.31	<0.001
platelet (10^9^/L)	198.61±56.34	209.49±51.73	<0.001
Neutrophil (10^9^/L)	4.08±1.55	3.46±0.99	<0.001
Lymphocyte (10^9^/L)	1.57±0.52	1.92±0.64	<0.001
Monocyte (10^9^/L)	0.39±0.15	0.37±0.14	<0.001
NLR	2.85±1.94	1.98±0.86	<0.001
PLR	138.05±74.46	118.64±43.60	<0.001
LMR	4.34±1.68	5.78±2.23	<0.001

Abbreviations: PACG: primary angle closure glaucoma. WBC: white blood cells. NLR: neutrophil-to-lymphocyte ratio. PLR: platelet-to-lymphocyte ratio. LMR: lymphocyte-to-monocytes ratio. Data are expressed as mean± standard deviation (SD). Chi-square test and independent student’s t-test was used.

### The ROC analyses of the studied variables

**Figure 1:**
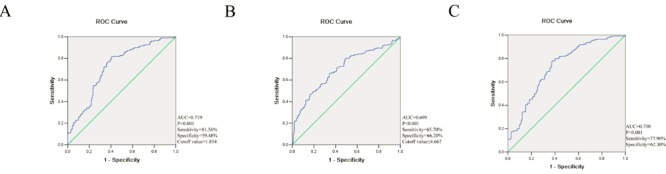
Receiver operating characteristics curve (ROC) analysis for neutrophil to lymphocyte ratio (NLR) **A.**, lymphocyte to monocyte ratio (LMR) **B.** and NLR+LMR **C.** in predicting primary angle closure glaucoma. AUC = area under the curve.

The ROC analyses of the studied variables are shown in Figure 1. According to this, the AUROC (area under the ROC) value of the NLR and LMR to distinguish patients (PACG, *n* = 771) and control subjects (*n* = 770) was found to be 0.719, 0.699, respectively. The best cutoff value was 1.854, 4.667, with a sensitivity of 81.56%, 65.7% and a specificity of 59.48%, 66.2%, respectively (Figure 1A and 1B). Moreover, the AUROC value of the NLR+ LMR was found to be 0.730, with a sensitivity of 77.9% and a specificity of 62.3% (Figure 1C).

### Comparison of laboratory parameters and ocular parameters in subjects with PACG, stratified according to severity

39 mild PACG subjects were excluded as they could not be age and sex matched to the moderate and severe PACG group in this section. Based on the MD, the PACG subjects were categorized into 3 subgroups of different severity level of which 183 were classified as mild, 174 as moderate and 375 as severe. There was no statistical difference in the mean age (*p* = 0.178) and gender (*p* = 0.248) among the three groups. The mean levels of neutrophil, NLR and WBC was lowest in the mild PACG group, followed by moderate PACG and severe PACG, and the differences among groups were significant (*p* = 0.003, *p* = 0.001, *p* = 0.006, respectively). The moderate PACG subgroup had a higher level of platelets than severe PACG (*p* = 0.033). Moreover, the IOP (*p* < 0.001), VCDR (vertical cup-disc ratio) (*p* < 0.001), and MD (*p* < 0.001) were greatest in the severe PACG group. The MS (visual fields mean sensitivity) was smaller in the severe PACG group (*p* < 0.001). Detailed information are shown in Table 2.

**Table 2:** Comparison of laboratory parameters and ocular parameters in subjects with PACG, stratified according to severity

Factors	Mild PACG, *n*= 183	Moderate PACG, *n*= 174	Severe PACG, *n*= 375	*P* value
Age, years	63.36±7.97	65.11±9.837	63.58±10.99	0.178
Gender (Male/Female)	62/121	53/121	141/234	0.248
IOP (mm Hg)	27.90±10.20	27.17±9.31	34.92±13.10	<0.001^a,c^
VCDR	0.45±0.17	0.50±0.20	0.72±0.24	<0.001^a,b,c^
CCT (μm)	539.11±39.16	549.55±61.35	549.62±46.49	0.108
ACD (mm)	1.85±0.36	1.85±0.44	1.87±0.76	0.893
AL (mm)	22.31±0.97	22.33±1.02	22.50±1.50	0.223
MD (dB)	3.335±1.61	8.73±1.83	21.70±5.61	<0.001^a,b,c^
MS (dB)	22.06±5.15	18.12±2.33	6.94±5.32	<0.001^a,b,c^
WBC (10^9^/L)	5.87±1.76	6.03±1.63	6.38±2.01	0.006a,c
platelet (10^9^/L)	194.39±53.76	207.50±57.74	194.91±56.82	0.033^b,c^
Neutrophil (10^9^/L)	3.81±1.43	3.95±1.21	4.25±1.68	0.003^a,c^
Lymphocyte (10^9^/L)	1.56±0.46	1.59±0.55	1.56±0.52	0.780
Monocyte (10^9^/L)	0.38±0.14	0.39±0.13	0.40±0.16	0.219
NLR	2.58±1.09	2.62±0.87	2.98±1.55	0.001^a,c^
PLR	132.28±45.23	148.21±120.54	135.81±57.95	0.103
LMR	4.40±1.52	4.42±1.55	4.26±1.81	0.485

Abbreviations: IOP: intraocular pressure, VCDR: vertical cup-disc ratio, CCT: central corneal thickness, AL: axial length, ACD: anterior chamber depth, MD: visual fields mean deviation, MS: visual fields mean sensitivity, PACG: primary angle closure glaucoma. WBC: white blood cells. NLR: neutrophil-to-lymphocyte ratio. PLR: platelet-to-lymphocyte ratio. LMR: lymphocyte-to-monocyte ratio. Data are expressed as mean± standard deviation (SD). Chi-square test and One-way ANOVA was used.

^a^P<0.05 for the difference between Mild PACG and Severe PACG (1-way ANOVA with the LSD post hoc test).

^b^P<0.05 for the difference between Mild PACG and Moderate PACG (1-way ANOVA with the LSD post hoc test).

^c^P<0.05 for the difference between Moderate PACG and Severe PACG (1-way ANOVA with the LSD post hoc test).

**Figure 2:**
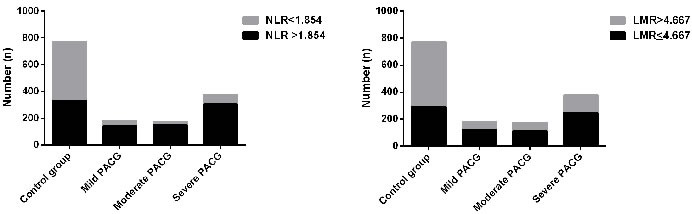
The number of patients with different PACG severity in two groups stratified by neutrophil to lymphocyte ratio (NLR) **A**. and lymphocyte to monocyte ratio (LMR) **B.**

Based on the best cutoff value of NLR and LMR, the subjects were divided into two groups (NLR > 1.854 group, NLR ≤ 1.854 group; LMR ≤ 4.667 group, LMR > 4.667 group, respectively) in mild PACG, moderate PACG, severe PACG, and control group, respectively. As shown in Table 3 and Figure 2, the proportion of subjects in PACG was higher in the NLR > 1.854 group than that of the control group (*p* < 0.001). Similarly, the proportion of subjects in PACG was higher in the LMR ≤ 4.667 group than that of the control group (*p* < 0.001).

**Table 3:** The number of subjects in different group, according to NLR and LMR

	Mild PACG	Moderate PACG	Severe PACG	Control group	*P* value
NLR					
NLR >1.854	142	151	305	328	
NLR≤1.854	41	23	70	442	^a, b, c^<0.001
LMR					
LMR ≤4.667	122	111	247	293	
LMR>4.667	61	63	128	477	^a, b, c^<0.001

Abbreviations: PACG: primary angle closure glaucoma. NLR: neutrophil-to-lymphocyte ratio. LMR: lymphocyte-to- monocyte ratio. Chi-square test was used.

^a^P<0.05 for the difference between Mild PACG and Control group.

^b^P<0.05 for the difference between Moderate PACG and Control group.

^c^P<0.05 for the difference between Severe PACG and Control group.

### Pearson correlation for associations between laboratory parameters and glaucoma severity in PACG

**Figure 3:**
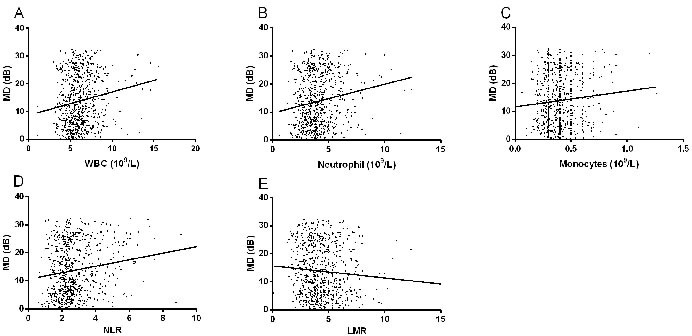
Scatterplot of patient individual measurements for white blood cell (WBC) **A.**, neutrophil **B.**, monocyte **C.**, neutrophil to lymphocyte ratio (NLR) **D.**, and lymphocyte to monocyte ratio (LMR) **E.** *versus* MD (visual fields mean deviation); each data point represents one patient.

Pearson correlation analysis revealed significant correlations between VCDR and NLR (r = 0.245, *p* < 0.001), as well as between MD and NLR (r = 0.175, *p* < 0.001) in the PACG group, as shown in Table 3 and Figure 3. The correlation between WBC, neutrophil, monocyte, and LMR with glaucoma severity were also significant, WBC and VCDR (r = 0.175, *p* < 0.001), WBC and MD (r = 0.179, *p* < 0.001), neutrophil and IOP (r = 0.076, *p* = 0.036), neutrophil and VCDR (r = 0.242, *p* < 0.001), neutrophil and MD (r = 0.184, *p* < 0.001), monocyte and MD (r = 0.092, *p* = 0.017), LMR and MD (r = −0.080, *p* = 0.038). (Table 4 and Figure 3)

**Table 4:** Pearson correlation between laboratory parameters and glaucoma severity in primary angle closure glaucoma

Factors	IOP	VCDR	MD
WBC	NS	r = 0.175, p < 0.001	r = 0.179, p < 0.001
Neutrophil	r = 0.076, p = 0.036	r = 0.242, p < 0.001	r = 0.184, p < 0.001
Monocytes	NS	r = 0.080, p = 0.036	r = 0.092, p = 0.017
NLR	NS	r = 0.245, p < 0.001	r = 0.175, p < 0.001
LMR	NS	r = −0.174, p < 0.001	r = −0.080, p = 0.038

Abbreviations: IOP: intraocular pressure. VCDR: vertical cup-disc ratio. MD: visual fields mean deviation. WBC: white blood cells. NLR: neutrophil-to-lymphocyte ratio. LMR: lymphocyte-to-monocyte ratio. NS: not significant.

### Multiple linear regressions for associations between laboratory parameters and glaucoma severity in PACG

Table 5 demonstrated the multiple linear regressions of WBC, neutrophil, NLR, and LMR with the ocular parameters. In multiple regression analysis after adjusting for IOP, CCT (central corneal thickness), ACD (anterior chamber depth), and AL (axial length), there was a significant correlation between WBC and MD (B = 0.151, *p* < 0.001), neutrophil and MD (B = 0.143, *p* = 0.003), NLR and MD (B = 0.144, *p* = 0.001), LMR and MD (B = −0.100, *p* = 0.034).

**Table 5:** Multiple linear regressions for associations between laboratory parameters and glaucoma severity in primary angle closure glaucoma

Factors	VCDR B (*P* value, 95%CI)	MD B (*P* value, 95%CI)
WBC	0.164 (<0.001, −0.033 to 0.010)	0.151 (0.001, 0.307 to 1.266)
Neutrophil	0.209 (<0.001, −0.046 to 0.018)	0.143 (0.003, 0.307 to 1.436)
NLR	0.222 (<0.001, −0.054 to 0.023)	0.144 (0.002, 0.360 to 1.602)
LMR	0.071 (0.135, −0.003 to 0.023)	−0.100 (0.034, −1.034 to −0.041)

Abbreviations: VCDR: vertical cup-disc ratio. MD: visual fields mean deviation. WBC: white blood cell. NLR: neutrophil-to-lymphocyte ratio. LMR: lymphocyte-to- monocyte ratio. Adjusted for age, gender, intraocular pressure, central corneal thickness, axial length, and anterior chamber depth.

### The association of laboratory parameters with PACG and control individuals by logistic regression analysis

Logistic regression analyses were performed to identify the independent risk factors for PACG compared with control subjects (Table 6). Logistic regression analyses revealed that WBC (OR = 1.208, 95%CI = 1.179 to 1.238), neutrophil (OR = 1.598, 95%CI = 1.541 to 1.656), NLR (OR = 2.313, 95%CI = 2.200 to 2.431), and LMR (OR = 0.682, 95%CI = 0.666 to 0.699) were associated with PACG after adjusting for age and sex.

**Table 6:** The association of laboratory parameters with PACG and control individuals by logistic regression analysis

Factors	OR	*p* value	95%CI
Age	1.002	0.372	0.998 to 1.006
Gender	0.914	0.401	0.741 to 1.128
WBC (10^9^/L)	1.208	<0.001	1.179 to 1.238
Neutrophil (10^9^/L)	1.598	<0.001	1.541 to 1.656
NLR	2.313	<0.001	2.200 to 2.431
LMR	0.682	<0.001	0.666 to 0.699

Abbreviations: WBC: white blood cell. NLR: neutrophil-to-lymphocyte ratio. LMR: lymphocyte-to- monocyte ratio. Adjusted for age, and gender.

### Nomogram for predicting PACG

**Figure 4:**
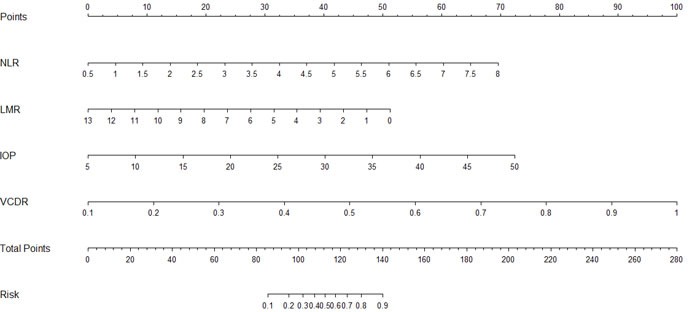
Nomogram for predicting primary angle closure glaucoma NLR: neutrophil-to-lymphocyte ratio. LMR: lymphocyte-to-monocytes ratio. IOP: intraocular pressure. VCDR: vertical cup-disc ratio.

The risk factors that were predictive of PACG were as follows: IOP, VCDR, NLR, and LMR. A nomogram to predict risk factors for PACG was developed (Figure 4). As IOP, VCDR, NLR, and LMR were independent risk factors for PACG, these variables were included in the nomogram.

## DISCUSSION

This study for the first time reported that patients with PACG have increased WBC, neutrophil, monocyte, NLR, and PLR levels, and have decreased platelet, lymphocyte and LMR levels compared with controls. In addition, WBC, neutrophil, NLR and LMR were significantly associated with VCDR and MD, both indicators of glaucomatous visual field defects. Furthermore, ROC analysis showed that the AUROC value of the NLR and LMR to distinguish PACG patients and control subjects was found to be 0.719, 0.699, with best cutoff values of 1.854 and 4.667, respectively.

Based on the evidence provided in a series of studies, autoimmune and/or active infection-induced inflammation was associated with the onset and/or development of glaucoma [3–8]. Leibovitch et al. [16] suggested that a C-reactive protein level was higher in normal tension glaucoma patients than normal controls. Similar results as those mentioned above were also reported by de Voogd et al. in open-angle glaucoma [17], and Mocan et al. [18] in exfoliative glaucoma. Moreover, the concentration of proinflammatory cytokine profile (IL-6, IL-1β, TGF-β1, IL-8, and so on) in the aqueous humor from glaucomatous eyes was also higher than controls [19, 20], as well as levels of proinflammatory cytokine from peripheral blood [21], which suggests that increased levels of these cytokines were associated with activation of leukocytes in glaucomatous eyes. Wong et al. [22] observed an upward trend in the expression of IL-2 and IFN-γ in the iris of primary open angle glaucoma and chronic angle closure glaucoma patients. In this study, we also found that patients with PACG have increased WBC, neutrophil, monocyte, NLR, and PLR levels compared with controls. All of these findings revealed that glaucoma may be associated with inflammatory processes.

Research to uncover the exact inflammatory mechanism involved in the pathogenesis of PACG is still ongoing. Inflammatory episodes outside the eyes, such as those due to acute systemic infections, may be relevant to glaucoma. Astafurov et al. [8] reported that patients with glaucoma had higher bacterial oral counts compared to control subjects and lipopolysaccharide administration in glaucoma animal models resulted in enhancement of axonal degeneration and neuronal loss. In addition, Zeng et al. [7] performed a meta-analysis which showed a statistically significant association between H. pylori infection and open-angle glaucoma (OR = 2.08, 95% CI = 1.42-3.04). Active infection (higher bacterial oral counts, H. pylori infection) may result in higher levels of WBC and neutrophil in patients with PACG. During inflammation, several proinflammatory cytokine and acute phase proteins are released. However, in what way could inflammation cause PACG? One reason might be that inflammatory proteins and cells cause mechanical blockage or damage to the trabeculum, leading to increased intraocular pressure [23]. This suggests that proinflammatory cytokine and acute phase proteins might not only be a biomarker but an active mediator in the pathogenesis of PACG. However, this could not be demonstrated in this study.

We also explored the association between WBC, neutrophil, monocyte, NLR, and LMR levels with PACG severity, and found that the levels of WBC, neutrophil, and NLR was significantly and positively associated with PACG severity, and LMR was negatively associated with PACG severity. This suggested that an inflammatory mechanism was associated with the degeneration of retinal ganglion cells and their axons. Secondary neurodegeneration is thought to play an important role in the pathology of neurodegenerative disease. It has been recently proposed that C3-dependent microglial priming confers susceptibility in multiple sclerosis, resulting in microglial over activation in response to secondary insults [24]. Proinflammatory cytokine and acute phase proteins can lead to the activation of the complement system. Microglia activation is documented to play a role in secondary neurodegeneration in glaucoma [25]. As secondary degenerative events occur over a greater time-frame than primary degenerative events [26], this might explain how the levels of WBC, neutrophil, NLR and LMR were significantly associated with PACG severity. Moreover, the level of platelet was decreased when compared to controls in this study. The reason for the difference in platelet levels and it’s relationship with the etiopathogenesis of PACG might be due to the fact that inflammatory mediators and mitotic substances are released by activated platelets [27], and thus increase platelets and WBC in the ocular tissues. This suggests that the activation of platelets might play a role in the development of PACG. Monocytes are the main source of cytokines and play a pivotal role in inflammation [28]. The level of monocyte was also increased in patients with PACG, although it was not associated with PACG severity. The exact role of monocyte in PACG requires further research.

Currently, as a simple, rapid, and reliable parameter, NLR and LMR is recommended for predicting systemic inflammatory conditions, including primary open angle glaucoma [14], age-related macular degeneration [29], pseudoexfoliation glaucoma [15], pseudoexfoliation syndrome [30], and retinal vein occlusion [31]. For example, Ozgonul et al. [14] reported that NLR and PLR can be used as novel biomarkers in primary open angle glaucoma; Dursun et al. [31] suggested that the optimal cutoff value of NLR to predict retinal vein occlusion was 1.89, with 72.5% sensitivity and 100% specificity. All these results demonstrating the diagnostic value of NLR and LMR in ocular diseases implies that inflammatory cascades have critical roles in their pathophysiology. However, there are currently no studies reporting the diagnostic value of NLR and LMR in PACG. In this study, we found that the best cutoff value of NLR to distinguish PACG patients and control subjects was 1.854, with 81.56% sensitivity and 59.48% specificity. The best cutoff value of LMR was 4.667, with 65.7% sensitivity and 66.2% specificity. Furthermore, the AUROC value of the NLR+ LMR was found to be 0.730, with a sensitivity of 77.9% and a specificity of 62.3%. AUROC value of NLR and LMR were considered fair in this study, which was higher than those of the above studies. The best cutoff value is valuable to refer patients to more advanced tests for definite diagnosis. Based on the best cutoff value of NLR and LMR, the subjects were divided into two groups (NLR > 1.854 group, NLR ≤ 1.854 group; LMR ≤ 4.667 group, LMR > 4.667 group, respectively) in mild PACG, moderate PACG, severe PACG, and control group, respectively. We found that the proportion of subjects in PACG was higher in the NLR > 1.854 group than that of the control group (*p* < 0.001). Similarly, the proportion of subjects in PACG was higher in the LMR ≤ 4.667 group than that of the control group (*p* < 0.001) (Table 3 and Figure 3). Therefore, we believe that the cutoff value of NLR and LMR may have a crucial role in distinguishing PACG patients and control subjects. In simpler terms, it appears that both NLR and LMR may be novel biomarkers in PACG.

**Figure 5:**
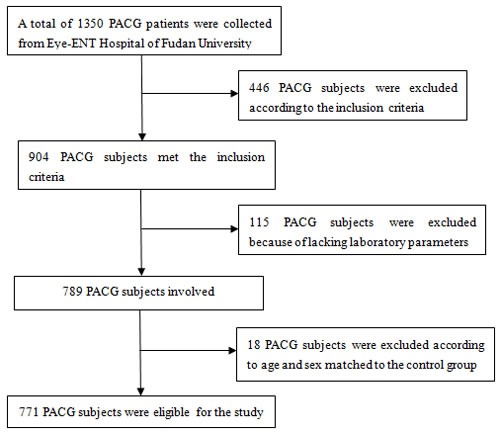
Flow chart of patients excluded from the study

Nomograms have been widely used for quantifying the risk factors of various diseases [32, 33]. The effects of several separate variables are integrated by a nomogram to give an individualized risk assessment for each patient. In this study, the patients with high IOP, large VCDR, increased NLR, and decreased LMR, were in the high-risk of PACG, which was shown in Figure 5. For example, a patient with IOP = 20 (25 points), VCDR = 0.5 (45 points), NLR = 3.5 (28 points), and LMR = 8 (18 points) would score 116 total points that converts to a risk probability for PACG of 50%.

To the best of our knowledge, this is the first report exploring the association between WBC, neutrophil, NLR, and LMR in patients with PACG. However, our study had some limitations. First, owing to this study being a retrospective case-control study, information on ocular parameters in controls subjects was lacking. Second, the data were collected at a single eye center, which may limit the generalizability of the results. Lastly, those on treatment with agents affecting laboratory parameters were excluded, but we did not consider treatment with brinzolamide eye drops, carbonic anhydrase inhibitor and mannitol and how this may affect the results.

In conclusion, our study provides findings that WBC, neutrophil, NLR, and LMR are independently related with PACG, and NLR and LMR may be useful as biomarkers in patients with PACG. These results suggest that systemic inflammatory response is involved in the onset and/or development of PACG.

## MATERIALS AND METHODS

### Patients

A retrospective review of 771 patients with newly and referral diagnosed PACG, was carried out between January 2010 to December 2015 from Eye-ENT Hospital of Fudan University. 770 age-matched and sex-matched normal controls were consecutively recruited from subjects who participated in yearly health screenings during the study period. The research was approved by the Ethics Committee of the Eye-ENT Hospital of Fudan University, Shanghai, China and was conducted according to the Declaration of Helsinki. Inform consent was obtained for all patients at the time of admission to the Eye-ENT Hospital of Fudan University. The study cohort flow diagram is shown in Figure 5. Each PACG patients underwent a standardized ophthalmic examination, which included refractive status, slit-lamp biomicroscopy, fundus examination, IOP (intraocular pressure), CCT (central corneal thickness), AL (axial length), ACD (anterior chamber depth), visual field examination, and gonioscopy, performed by glaucoma specialists. MD and MS were measured with the Octopus automated perimetry (HAAG, STREIT, Switzerland). IOP was measured using Goldmann applanation tonometry. Fundus photography was performed with a retinal camera (TRC-NW200, Topcon). A-scan ultrasound (A-Scan Pachymeter, Ultrasonic, Exton, PA, USA) was used to measure AL, ACD, and CCT. Moreover, medical examinations were performed by respective physicians for all subjects at the Eye-ENT Hospital of Fudan University. Clinical and demographic information were obtained from the medical data platform of Eye-ENT Hospital of Fudan University by trained staff (SJ Li and BH Tang) using standardized data collection and quality-control procedures, resulting in reliable data for analysis. This database was used previously to study patients with glaucoma [34].

### Inclusion criteria

Our previous study has described the inclusion criteria of PACG [34] which are detailed below.

(1) PACG subjects were selected from inpatients.

(2) PACG was diagnosed on the basis of narrow angles with glaucomatous optic neuropathy with corresponding visual field loss. This was defined as glaucoma hemifield test outside normal limits including a cluster of three or more, non-edge, contiguous points on the pattern deviation plot, not crossing the horizontal meridian with a probability of less than 5% of being present in the age-matched normal (one of which was less than1%), an abnormal pattern standard deviation with a P value less than 5% occurring in the normal population, and fulfilling the test reliability criteria (fixation losses less than 20%, false positives less than 33% and/or false negatives less than 33%). PACG was diagnosed in eyes with narrow angles, with elevated IOP (IOP > 21 mm Hg); at least 180 degree of angle-closure obliterating the pigmented part of the trabecular meshwork, whether synechial or appositional, segmented or continuous; and eyes in which the degree of peripheral anterior synechial is too extensive to be managed by laser peripheral iridotomy. [35–37] Subjects receiving glaucoma medications were also included. Patients were divided into three groups with different severity based on perimetry, i.e. mild (MD≤6.00 dB), moderate (12 dB ≥ MD > 6 dB) and severe (MD > 12 dB) [38, 39]. 39 mild PACG subjects were excluded according to age and sex matched to the moderate and severe PACG group in the subgroup analysis.

(3) PACG subjects had no other ocular diseases, active infection and major systemic diseases (acute inflammation, chronic inflammation, hyperuricemia, diabetes, cardiac, autoimmune disease and cancer) that could probably affect the platelets, WBC, neutrophil, lymphocyte, monocytes levels. Active infection was defined as patients with fever (> 38°C) in this study [40], in order to obtain reliable data for analysis.

(4) Because this is a retrospective study, systemic diseases and medications of control subjects were both self-reported. Control subjects had no other ocular diseases, active infection and major systemic diseases (acute inflammation, chronic inflammation, hyperuricemia, diabetes, cardiac, autoimmune disease and cancer) that could probably affect the platelets, WBC, neutrophil, lymphocyte, monocytes levels. Control subjects were also excluded if there was any family history of glaucoma (self-reported). 430 normal control subjects were excluded based on the inclusion criteria. The exclusion rate of the control group was 35.75%. A total of 770 control subjects were eligible for the study.

### Laboratory analysis

In this study, PACG patients were selected from the hospital inpatients. As part of a standard care at the Fudan’s University Eye & ENT Hospital, peripheral blood samples were routinely collected from all the inpatients and tested for complete blood count. Complete blood counts, which included platelet, WBC, neutrophil, lymphocyte, monocyte were measured with the Mindray BC-5500 (Shenzhen, China) automatic blood counting system. All blood samples in our study were collected in ethylenediaminetetraacetic acid (EDTA) tubes. Laboratory parameters measurements were performed within 0.5 hours after blood collection. Internal controls were analyzed daily over the 5-year period, with no significant changes in the values. The NLR, PLR and LMR were calculated as the ratio of the neutrophil to lymphocyte, platelet to lymphocyte and that of lymphocyte to monocyte. Reference values for platelet, WBC, neutrophil, lymphocyte, and monocyte were 100-300*10^9^/L, 4-10*10^9^/L, 2-7*10^9^/L, 0.8-4*10^9^/L, and 0.1-0.8*10^9^/L, respectively.

### Statistics analysis

The data was analyzed using SPSS13.0 (SPSS Inc., Chicago, IL). Results are presented as mean±standard deviation (SD). Normality was assessed with the Kolmogorov-Smirnoff test. The independent student’s *t*-test and chi-square test were used for comparison of characteristics of patients between the groups. The one-way ANOVA test was used to compare the levels of laboratory parameters and ocular parameters among the three groups. The associations between laboratory parameters and ocular parameters in PACG were analysed using Pearson correlation. After Pearson correlation, multivariate linear regression analysis was performed to evaluate the association between WBC, neutrophil, NLR, LMR and disease severity, namely visual field indices (MD), VCDR, and other ocular parameters. Receiver operating characteristics (ROC) analysis was performed to demonstrate the optimal cutoff value for predicting PACG, and then these cutoff values were used to calculate the sensitivity and specificity. Binary logistic regression models were developed to identify the probabilities of the NLR, LMR, ROC analysis was then performed to demonstrate the sensitivity and specificity of the probabilities of the NLR+LMR for predicting PACG. Upon the verification of the prediction value of NLR and LMR as a continuous variable, we further investigated the severity relevance of the categorized NLR and LMR that assign patients into two groups base on the cutoff value (NLR > 1.854, NLR ≤ 1.854; LMR ≤ 4.667, LMR > 4.667). Areas under the ROC ≥ 0.90-1.00 were considered excellent, ≥ 0.80-90 considered good, ≥ 0.70-80 considered fair, ≥ 0.60-0.70 considered poor and < 0.50-60 fail [41]. Logistic regression analyses were performed to identify the independent risk factors for PACG (control group = 0, PACG = 1; male = 0, female = 1). Odds ratios (ORs) with 95% confidence intervals (95%CIs) were estimated by Logistic regression analyses. The nomogram was constructed based on the logistic regression model using the R project for statistical computing (R version 3.3.2). A two-sided P < 0.05 was considered statistically significant.
